# Dissipative Particle Dynamics Study on Interfacial Properties of Symmetric Ternary Polymeric Blends

**DOI:** 10.3390/polym13091516

**Published:** 2021-05-08

**Authors:** Dongmei Liu, Kai Gong, Ye Lin, Tao Liu, Yu Liu, Xiaozheng Duan

**Affiliations:** 1 School of Science, North China University of Science and Technology, Tangshan 063210, China; gongkai0524@163.com (K.G.); linye315317@163.com (Y.L.); vampireliu@outlook.com (Y.L.); 2 State Key Laboratory of Polymer Physics and Chemistry, Changchun Institute of Applied Chemistry, Chinese Academy of Sciences, Changchun 130022, China; 3 State Key Laboratory of Molecular Engineering of Polymers, Department of Macromolecular Science, Fudan University, Shanghai 200438, China

**Keywords:** dissipative particle dynamics, interfacial property, interfacial tension, compatibilizer

## Abstract

We investigated the interfacial properties of symmetric ternary *A*_n_/*A*_m_*B*_m_/*B*_n_ and *A*_n_/*A*_m/2_*B*_m_*A*_m/2_/*B*_n_ polymeric blends by means of dissipative particle dynamics (DPD) simulations. We systematically analyzed the effects of composition, chain length, and concentration of the copolymers on the interfacial tensions, interfacial widths, and the structures of each polymer component in the blends. Our simulations show that: (i) the efficiency of the copolymers in reducing the interfacial tension is highly dependent on their compositions. The triblock copolymers are more effective in reducing the interfacial tension compared to that of the diblock copolymers at the same chain length and concentration; (ii) the interfacial tension of the blends increases with increases in the triblock copolymer chain length, which indicates that the triblock copolymers with a shorter chain length exhibit a better performance as the compatibilizers compared to that of their counterparts with longer chain lengths; and (iii) elevating the triblock copolymer concentration can promote copolymer enrichment at the center of the interface, which enlarges the width of the phase interfaces and reduces the interfacial tension. These findings illustrate the correlations between the efficiency of copolymer compatibilizers and their detailed molecular parameters.

## 1. Introduction

Polymeric blends and mixtures have broad applications in many areas of science and technology. [1] In a polymer blend, the chemically different polymers often exhibit immiscibility; and consequently, the mixture tends to phase-separate into macroscopic domains, resulting in the relatively poor mechanical properties of the materials [2]. In these immiscible polymer blends, the added amphiphilic compatible agents can selectively segregate at the interface [3] between the immiscible homopolymers, which improves the miscibility [4] between different types of polymers by reducing interfacial tension and promoting interfacial adhesion [5]. With this modification, the stabilized blends usually exhibit desired properties and performance [6,7]. Typically, the compatibilizers or emulsifying agents are block copolymers, such as diblock, triblock, random, and gradient copolymers containing segments that can be miscible with the homopolymers [7]. Among others, the simplest symmetric *A*_n_*B*_n_ diblock copolymer was widely used to optimize the interfacial properties. Moreover, the triblock copolymers were demonstrated to be more effective as compatibilizers in reducing the interfacial tension than diblock copolymers, as shown by the pioneering work of Russell et al. [8]

Experimental studies of triblock copolymers at the immiscible homopolymer interface were extensively performed [9,10,11,12,13,14,15,16,17]. For example, Wolf et al. [9] initially reported that interfacial tension rapidly decreases with the addition of poly(dimethyl siloxane)-poly(ethylene oxide)-poly(dimethyl siloxane) (PDMS-PEO-PDMS) triblock copolymers into the PDMS/PEO blends. Subsequently, they investigated the dependence of interfacial tension reduction on the copolymer architecture (PDMS-PEO diblock copolymers and PDMS-PEO-PDMS triblock copolymers) for the PDMS/PEO blend, which showed that the compatibilization efficiency of the copolymer was mainly dependent on the total number of PDMS segments for the range of molecular weights examined, and has little correlation with the copolymer architecture and the length of the PEO block [10]. Xu et al. reported that poly(methyl methacrylate)-polyethylene -poly(methyl methacrylate) (PMMA-PE-PPMA) can not only significantly improve the morphology of poly(methyl methacrylate)/polyethylene (PE/PMMA) (80/20) blends, but also enhance their elastic modulus and hardness [16]. Sun et al. studied the effects of polylactic acid Poly (butyleneadipate-co-terephthalate)-polylactic acid compatibilizers with two different molecular weights on immiscible polylactic acid/Poly (butyleneadipate-co- terephthalate) blends [17]. The thermal, morphological, and rheological analysis showed that the addition of triblock copolymers enhanced the miscibility and interfacial bonding strength between polylactic acid and Poly (butyleneadipate -co-terephthalate). Recently, Zhao et al. investigated the effects of styrene-ethylene/butylene-styrene on the interfacial tension of immiscible polypropylene/polystyrene mixture [18]. They found that the triblock copolymer compatibilizers with a shorter chain length perform at a higher efficiency than their long-chain length counterparts.

With computational technology’s rapid development, computer simulations serve as an important strategy to fundamentally analyze the structural and thermodynamic properties of the polymer blends [19,20]. For example, the Monte Carlo simulation was widely used to study the aggregation behavior of the *A*_n_/*A*_m_*B*_m_/*B*_n_ polymeric blends [21,22,23,24,25]. Balazs et al. [21], Wang et al. [22], and Muller et al. [23,24,25] reported a series of lattice Monte Carlo simulations on the interfacial properties between two immiscible polymer blends with and without compatibilizers, which showed the detailed conformational properties of polymers at the immiscible homopolymer interface. In the previous study, we utilized Monte Carlo simulation to examine the effects of diblock copolymer on the interfacial properties of *A*_n_/*A*_m_*B*_m_/*B*_n_ polymeric blends [26,27], and illustrated that the interfacial width, density distribution, and orientation of the diblock copolymer strongly depended on the diblock copolymers’ chain properties. In recent years, dissipative particle dynamics (DPD) simulations became a vital tool for studying the physicochemical properties of polymeric blends, including phase behavior, dynamics, and morphology evolution [28,29,30,31,32,33,34,35,36,37,38,39,40]. Specifically, Groot et al. [41] employed DPD simulation to calculate the interfacial tension of immiscible polymer blends. Qian et al. studied the effect of the diblock copolymer AB on the interfacial properties of immiscible A/B homopolymer blends by DPD simulation [5]. Ginzburg et al. studied the interfacial tension of oil/water/surfactant blends via DPD simulation and self-consistent field theory, and the results showed a semiquantitative agreement between the two methods [42]. Hong et al. systematically investigated the interfacial and structural properties of oil/water interface with the presence of octenyl succinic anhydride (OSA) through DPD simulations and experimental analysis [43]. They found that the interfacial tension can be quickly reduced by adding OSA. Zhang et al. employed DPD simulation to study the properties of the glycerol trioleate/n-butanol/TX-100/[Bmim][BF4] blends system [44]. They proved that the means of DPD simulation can accurately and efficiently predict the properties and compositions of complex multicomponent blends, which play a vital role in the development of new multifunctional complex materials.

Previous simulation works mainly reported the structural properties of the *A*_n_/*A*_m_*B*_m_/*B*_n_ polymeric blend systems, whereas investigation of *A*_n_/*A*_m/2_*B*_m_*A*_m/2_/*B*_n_ polymeric blends systems remains limited. However, such investigations are necessary for understanding the microscopic mechanism for interfacial properties optimization, such as why triblock copolymer has a higher efficiency than diblock copolymer, and how triblock copolymers work as compatibilizers.

Here, we explore the interfacial properties of symmetric ternary *A*_n_/*A*_m_*B*_m_/*B*_n_ and *A*_n_/*A*_m/2_*B*_m_*A*_m/2_/*B*_n_ polymeric blends by means of DPD simulations. We first introduce the model and simulation details used in our work, and then systematically analyze the effects of composition (diblock and triblock), copolymer chain length, and copolymer concentration on the interfacial tension, density distribution of different beads, and detailed chain conformations of the copolymers. Our work elucidates the fundamental mechanism for the superiority of short triblock copolymers as the compatibilizer, in comparison with that of the longer chains or diblock counterparts. Further, we highlight the significance of copolymer concentration in maintaining the stability of the polymer blends. Finally, we summarize our results and offer some concluding remarks.

## 2. Methods

### 2.1. Model

The DPD method is a mesoscopic simulation technique [45,46]. In our coarse-grained simulation, each DPD “bead” (or particle) represents a polymer segment. The pairwise interaction in the simulation can be described by a soft potential, meaning that the beads can overlap considerably. The motion of the beads satisfies Newton’s equations of motion,

(1) $$\frac{dr_{i}}{dt}=v_{i};\;m_{i}\frac{dv_{i}}{dt}=f_{i}$$

where the vector $r_{i}$ and $v_{i}$ represent the position and velocity of the *i*th bead, respectively. The total force $f_{i}$ acting on a bead *i* is the sum of the conservative forces, the dissipative forces, the random forces, and the harmonic spring force [41].

(2) $$f_{i}=\underset{j\neq i}{\sum }\left(F_{\mathrm{ij}}^{C}+F_{\mathrm{ij}}^{D}+F_{\mathrm{ij}}^{R}\right)+F_{i}^{S}$$

The conservative force $F_{\mathrm{ij}}^{C}$, the dissipative force $F_{\mathrm{ij}}^{D}$, the random force $F_{\mathrm{ij}}^{R}$, and the harmonic spring force $F_{i}^{S}$ are given by the following equation:

(3) $$F_{\mathrm{ij}}^{C}=-\alpha_{\mathrm{ij}}\omega^{C}\left(r_{\mathrm{ij}}\right)e_{\mathrm{ij}}$$

(4) $$F_{\mathrm{ij}}^{D}=-\gamma \omega^{D}\left(r_{\mathrm{ij}}\right)\left(v_{\mathrm{ij}}\cdot e_{\mathrm{ij}}\right)e_{\mathrm{ij}}$$

(5) $$\;F_{\mathrm{ij}}^{R}=\sigma \omega^{R}\left(r_{\mathrm{ij}}\right)\xi_{\mathrm{ij}}\Delta t^{-1/2}e_{\mathrm{ij}}$$

(6) $$F_{i}^{S}=\sum Cr_{\mathrm{ij}}$$

in which $r_{\mathrm{ij}}=r_{i}-r_{j}$, $r_{\mathrm{ij}}=\left|r_{\mathrm{ij}}\right|$, $e_{\mathrm{ij}}=r_{\mathrm{ij}}/r_{\mathrm{ij}}$, $v_{\mathrm{ij}}=v_{i}-v_{j}$. The repulsion parameter $\alpha_{\mathrm{ij}}$ is a constant that can describe the maximum repulsion between the interacting beads. γ, σ and $\xi_{\mathrm{ij}}$ are the friction coefficient, amplitude of the noise, and Gaussian random number with zero mean and unit variance, respectively. $\omega^{C}$, $\omega^{D}$, and $\omega^{R}$ denote the weight functions of the conservative force $F_{\mathrm{ij}}^{C}$, dissipative force $F_{\mathrm{ij}}^{D}$, and random force $F_{\mathrm{ij}}^{R}$, respectively. For the conservative force$\;F_{\mathrm{ij}}^{C}$, we simply take $\omega^{C}\left(r_{\mathrm{ij}}\right)=1-r_{\mathrm{ij}}$ for $r_{\mathrm{ij}}<1$ and $\omega^{C}\left(r_{\mathrm{ij}}\right)=0$ for $r_{\mathrm{ij}}\geq 1$. Unlike $\omega^{C}\left(r_{\mathrm{ij}}\right),$ $\omega^{D}\left(r_{\mathrm{ij}}\right)$ and $\omega^{R}\left(r_{\mathrm{ij}}\right)$ have a certain relation, which obeys the fluctuation-dissipation theorem, [41]

(7) $$\omega^{D}\left(r\right)={\left[\omega^{R}\left(r\right)\right]}^{2},\;\sigma^{2}=2\gamma k_{B}T$$

where $k_{B}\;$represents the Boltzmann constant and *T* is the temperature. The weight functions $\omega^{D}$, and $\omega^{R}$ can be simply chosen as the previous work of Groot and Warren [41].

(8) $$\omega^{D}\left(r\right)={\left[\omega^{R}\left(r\right)\right]}^{2}=\{\begin{array}{c} \begin{array}{cc} {\left(1-r\right)}^{2} & \left(r<1\right) \end{array}\\ \begin{array}{cc} 0 & \left(r\geq 1\right) \end{array} \end{array}$$

The conservative interaction $\alpha_{\mathrm{AB}}$ (A and B are two different types of beads) strength is proportional to Flory–Huggins parameters χ for polymers, and obey the following relationship [41].

(9) $$\alpha_{\mathrm{AB}}\approx \alpha_{\mathrm{AA}}+3.50\chi_{\mathrm{AB}}$$

The interaction parameter between the same type of beads is set as $\alpha_{\mathrm{AA}}=\alpha_{\mathrm{BB}}=25$. Further, the harmonic spring force in Equation (6) is employed to account for the connection between polymer beads, where *C =* 4.0 is the corresponding spring force constant.

### 2.2. Simulation Details

In the present work, we perform the DPD simulations in a cell of size 30 × 30 × 30 with periodic boundary conditions using the Materials Studio program (developed by Accelrys). The radius of interaction, the bead mass, and the temperature are chosen as the reduced unit $r_{c}=m=k_{B}T=1$ (where $r_{c}$ is the interaction radius and *m* is the bead mass). We set the number density of the beads as ρ=3 so that each simulation approximately includes 81,000 beads. The time step is taken as 0.05, and the friction coefficient γ is chosen as 4.5.

To investigate the effects of copolymer on interfacial properties, we include *A*_n_ homopolymers and *B*_n_ homopolymers as well as the diblock *A*_m_*B*_m_ or triblock *A*_m/2_*B*_m_*A*_m/2_ copolymers as the compatibilizers in the simulations. The chain length (*N*_cp_) of the diblock *A*_m_*B*_m_ and the triblock *A*_m/2_*B*_m_*A*_m/2_ are set as 8, 16, 28, 40, respectively. The copolymer concentration *c*_cp_ varies from 0.01 to 0.2. The chain length of the uniform homopolymers is fixed as $N_{A}=N_{B}=8$. The copolymer consists of the same number of *A* and *B* beads. We fixed the Flory–Huggins interaction parameters between different beads as $\chi_{\mathrm{AB}}=$ 4.298. As shown by Equation (9), the interaction parameter between *A* and *B* is set as $\alpha_{\mathrm{AB}}=\alpha_{\mathrm{AA}}+3.50\chi_{\mathrm{AB}}=40$, which leads to a regime of strong segregation of *A* and *B* [7]. In our simulation, we first perform 2.0 × 10^5^ steps, which were tested long enough for the system equilibration. In addition, we perform 5×10^4^ steps as the production runs. We carry out several parallel simulations and obtain the final results from the order of 10^3^ to 10^4^ statistically independent samples.

In this work, we not only studied the phase separation process of the blended system, but also the thermodynamic properties of the blended system. For the study on phase separation, we initially place molecular chains randomly in the simulation box and adopt $\chi_{\mathrm{ij}}=$ 0, i.e., $\alpha_{\mathrm{AA}}=\alpha_{\mathrm{BB}}=\alpha_{\mathrm{AB}}=25$ for 5 × 10^4^ steps to obtain initial homogenous mixtures of the systems.

The static structure factor *S*_AA_(*q*) of homopolymers *A*_n_ is calculated to identify phase properties,

(10) $$S_{\mathrm{AA}}\left(q\right)=\frac{1}{N_{A}}\overset{N_{A}}{\underset{i=1}{\sum }}\overset{N_{A}}{\underset{j=1}{\sum }}\langle e^{-iq\cdot \left(r_{i}-r_{j}\right)}\rangle$$

where $r_{i}$ and $r_{j}$ are the position vectors of beads *i* and *j* of homopolymer *A*_n_, $N_{A}$ is the total number of beads *A* in homopolymers, and *q* is the wave vector [47].

In a ternary blend with flat interfaces, one of the most important parameters is interfacial tension which can directly reflect interfacial properties. Also, the interfacial tension obtained from DPD simulation [35,36,37] is often used as a basis to compare with the theoretical treatment of Groot and Warren [41]. Likewise, we calculate the interfacial tension according to the Irving–Kirkood equation [48], which is obtained by integrating the stress difference over *x*,

(11) $$\gamma_{DPD}=\int \left[P_{\mathrm{xx}}-\frac{1}{2}\left(P_{\mathrm{yy}}+P_{\mathrm{zz}}\right)\right]dx$$

where *P* represents the pressure tensor, *x* is the axis normal to the interface, and *y* and *z* are the axes parallel to the interface. In addition, we obtain the orientation parameters by calculating the difference between the normal component and the transverse component of the mean-square radius of gyration <*R*_g_^2^> of the triblock copolymers according to the previous work of Qian et al. (Ref. [5]), which can be written as:

(12) $$q=\frac{\left(\langle R_{g}^{2}\rangle {}_{x}-1/2\left(\langle R_{g}^{2}\rangle {}_{y}+\langle R_{g}^{2}\rangle {}_{z}\right)\right)}{R_{g}^{2}}$$

where <*R*_g_^2^>_x_, <*R*_g_^2^>_y_, <*R*_g_^2^>_z_ are the components in three principal directions of <*R*_g_^2^>. We also calculate the mean-square end-to-end distance <*R*_ee_^2^> of the polymer chains to characterize the detailed polymer conformations.

## 3. Results and Discussion

### 3.1. Evolution of Phase Separation

**Figure 1:**
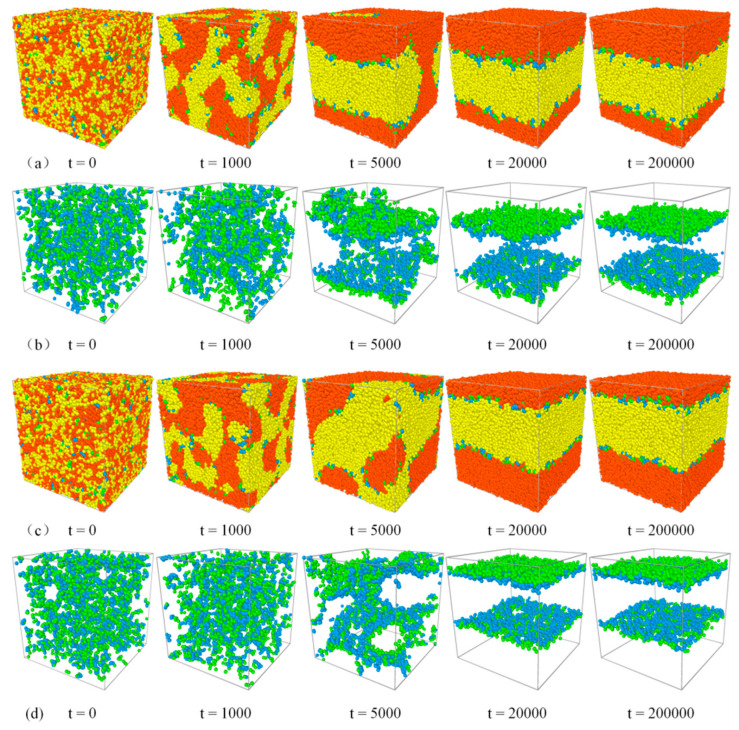
Representative morphology snapshots for ternary mixtures at different simulation times. Compositions are *A*_8_/*A*_4_*B*_4_/*B*_8_ for (**a**,**b**), and *A*_8_/*A*_2_*B*_4_*A*_2_/*B*_8_ for (**c**,**d**). Concentration of the copolymer is fixed as *c*_cp_ = 0.05. Red and yellow spheres represent bead *A* and bead *B* of homopolymers, and green and blue spheres represent beads *A* and *B* of the copolymers.

**Figure 2:**
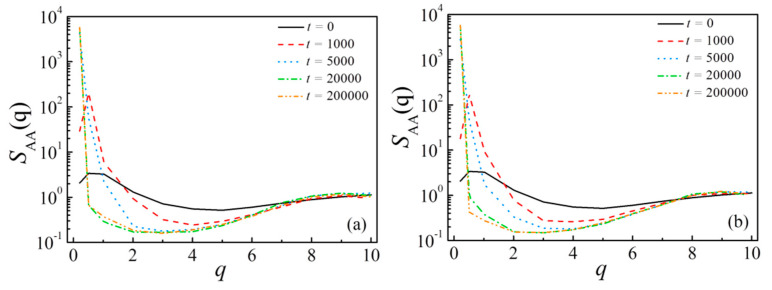
Structure factor of the beads *A* in homopolymer *A*_n_ for the cases of (**a**) *A*_8_/*A*_4_*B*_4_/*B*_8_ and (**b**) *A*_8_/*A*_2_*B*_4_*A*_2_/*B*_8_ at different simulation times. Copolymer concentration is set as *c*_cp_ = 0.05.

We start our analysis from the evolution of the blends’ phase separation in DPD simulation, in which the systems *A*_8_/*A*_4_*B*_4_/*B*_8_ and *A*_8_/*A*_2_*B*_4_*A*_2_/*B*_8_ are considered. Figure 1 and Figure 2 show the representative morphology snapshots and the structure-factor *S*_AA_(*q*) for the diblock and triblock systems at different simulation times (*t*), respectively. Here, the copolymer concentration is set as *c*_cp_ = 0.05. At *t* = 0, the copolymers and homopolymers were randomly mixed, and the blend exhibits a disordered phase state, corresponding to the black solid line of *S*_AA_(*q*) in Figure 2a,b. At *t* = 1000, as shown by the morphology and the characteristic peaks of *S*_AA_(*q*) at *q* < 3, we see the bicontinuous microemulsion (BME) structures of the blends, due to the immiscibility between beads *A* and *B*, which indicates the microscopic phase separation of the blends. With further time evolution, the smaller bicontinuous microemulsion gradually segregates into larger bicontinuous phases (at *t* = 5000), and the characteristic peaks of *S*_AA_(*q*) disappear. The system finally achieves the macrophase separation (at *t* >= 20,000), resulting in the formation of the *A* bulk phase and *B* bulk phase separated by the flat interfaces. In the final state, the structure factor *S*_AA_(*q*) obeys the Ornstein–Zernike form [49,50], and the maximum value of *S*_AA_(*q*) appears at the smallest *q* due to the macroscopic phase separation of the system, which also means that the size of the characteristic domain is infinite. Herein, *S*_AA_(*q*) is used to reflect the changes in the phase behaviors of the blend over time. We found that there is no substantial difference in *S*_AA_(*q*) between the systems with diblock and triblock copolymers, which is due to the similar macroscopic phase separation processes for *A*_8_/*A*_4_*B*_4_/*B*_8_ and *A*_8_/*A*_2_*B*_4_*A*_2_/*B*_8_ systems.

We also calculate the radial distribution functions between beads *A* and *B* [*g*_AB_(r)] from homopolymers *A*_n_ and *B*_n_, beads *A* and *A* [*g*_AA_(r)] of all homopolymers *A*_n_, beads *A* and *A* [*g*_AAintra_(r)] within homopolymers *A*_n_, and beads *A* and *A* [*g*_AAintrer_(r)] with different homopolymers *A*_n_ to characterize the detailed structural information of the blends during phase separation (as illustrated in Figures S1 and S2 in the Electronic Supplementary Information). In the initial state, homopolymers *A*_n_ and *B*_n_ are randomly mixed, which results in the small contact peaks of *g*_AB_(r) at r < 2 and the value of *g*_AB_(r) achieving a flat value of *g*_AB_(r) = 1 at large r. With the time evolution, due to the significant phase separation between homopolymers *A*_n_ and *B*_n_, the contacting between beads *A* and *B* is hindered, which results in the *g*_AB_(r) < 1 at the equilibrium state in Figure S1a,b. In accord, the corresponding value of *g*_AA_(r) exhibits a significant increase, which indicates the structural variations within the polymer blends. Note that in the *A*_n_/*A*_m/2_*B*_m_*A*_m/2_/*B*_n_ blends, the central *B* block of the triblock copolymer preferentially segregated into *B*_n_ phase, whereas the corresponding *A* end blocks segregated into the *A*_n_ phase, which implies the folding structure of the copolymers.

After verifying the blends‘ phase separation, we next initiated the structure of homopolymers and copolymers in the distinct of the box along the *x*-direction and performed the simulation [5]. This design serves as an effective strategy to speed up the formation of the interfaces (perpendicular to the *x*-direction) for the accurate calculation of the interfacial tension, and to save the computational cost [5].

### 3.2. Comparison between A_n_/A_m_B_m_/B_n_ and A_n_/A_m/2_B_m_A_m/2_/B_n_ Blends

**Figure 3:**
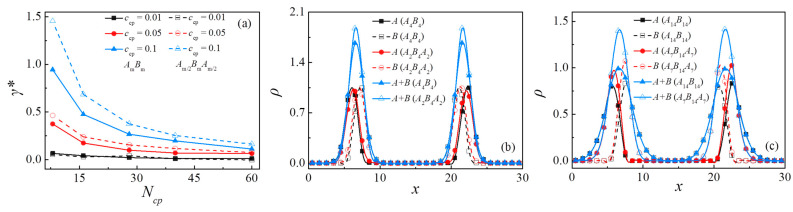
(**a**) Reduced interfacial tension $\gamma^{*}=\gamma_{0}-\gamma$ of blends as a function of copolymer chain length at copolymer concentration of *c*_cp_ = 0.01, 0.05, 0.1. Density profiles of beads *A*, *B,* and *A + B* of copolymer along the *x*-axis at (**b**) *N*_cp_ = 8 and (**c**) *N*_cp_ = 28, where *c*_cp_ = 0.1.

Due to the unique amphiphilic features, copolymers are usually used as compatibilizers to lower the interfacial tension between the immiscible homopolymers and enhance the thermal stability of the polymer mixtures. To examine the efficiency of diblock and triblock copolymers as the compatibilizers, we examine the interfacial tension of the two ternary systems. Figure 3a shows the reduced interfacial tension $\gamma^{*}=\gamma_{0}-\gamma$ (where γ and $\gamma_{0}$ indicate the interfacial tensions’ binary homopolymer mixtures with and without the copolymers) as a function of the chain length of the added diblock and triblock polymers at different copolymer concentrations.

In our simulations, it is noted that when the copolymer concentration is fixed as *c*_cp_ = 0.01, the interfacial tensions between diblock and triblock systems differ slightly, and the interfacial tension is almost unchanged. With the increase of copolymer concentration, there appears to be a significant discrepancy in the reduced tensions between blends containing the diblock and triblock copolymers; the reduced interfacial tension of the triblock polymer system is always higher than the diblock polymer system. Therefore, we can conclude that the triblock polymers serve as more effective compatibilizers than the diblock in reducing the interfacial tension. This result is supported by the experimental observations of Russell et al. [8]

To further elucidate the underlying mechanisms of interfacial tension reduction caused by copolymer addition, we calculate the relative density profiles for beads *A* and *B* of the homopolymers and copolymers along the *x*-axis. Figure 3b,c show the density distribution of beads *A*, *B*, and *A* + *B* of the diblock and the triblock. The chain lengths of the copolymers are set as *N*_cp_ = 8 and *N*_cp_ = 28, respectively; the concentration of the copolymer is *c*_cp_ = 0.1. As shown in these figures, all copolymer beads aggregate at the interface, with beads *A* and *B* penetrating the *A* and *B* homopolymer phases, respectively. For the case of *N*_cp_ = 8, the density of beads *A+B* of the triblock copolymers at the center of the interface (the vacant blue triangles) is slightly higher than that of the diblock (the solid blue triangles). As shown by Figure 3c, this phenomenon turns out to be more pronounced with the increase in copolymer chain length. Specifically, as the chain length of copolymer *N*_cp_ = 28, the *A* and *B* beads’ density of the triblock copolymers at the interface was also higher than that of the diblock copolymers, which indicates the enhanced enrichment of the triblock at the interface.

Since we demonstrated that the triblock copolymers exhibit better performance in maintaining the stability of the polymer mixture at the molecular level, we next focus on exploring the effects of chain length and concentration of the triblock copolymers on the phase properties of the polymer blends.

### 3.3. Effect of Chain Length of Triblock Copolymers

**Figure 4:**
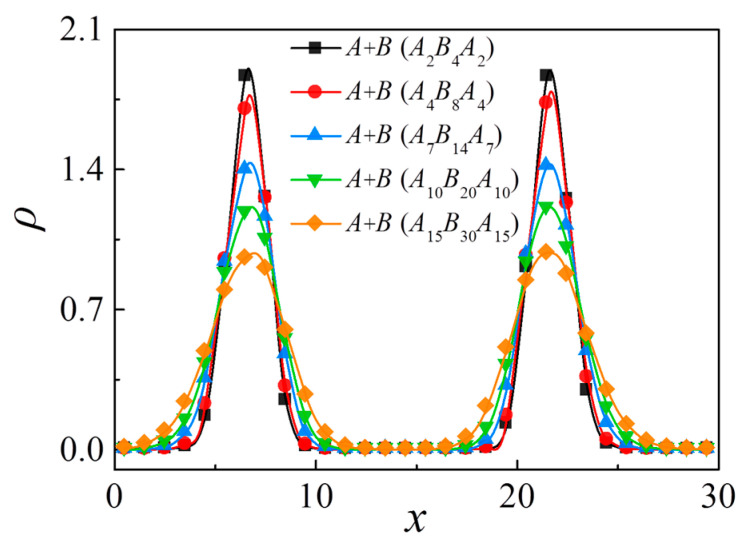
Density profiles of beads *A + B* of triblock copolymer along *x*-axis as a function of chain length of copolymer at copolymer concentration of *c*_cp_ = 0.1.

In Figure 3a, we found that for the polymer blends composed of *A*_n_/*A*_m/2_*B*_m_*A*_m/2_/*B*_n_, the reduced interfacial tension of the blends exhibits a significant decay with increasing the chain length of triblock copolymers; that is, the triblock copolymer compatibilizers with a shorter chain length show a higher efficiency in maintaining the stability of the blends than their counterparts with longer chain length. This finding is consistent with the experimental studies of Zhao et al. [18]. More specifically, Figure 4 shows the relative density profiles of triblock copolymers with different chain lengths at a fixed concentration of *c*_cp_ = 0.1, which illustrates that the density of beads *A+B* of the triblock copolymer at the center of the interface decreases with increases in the triblock copolymer chain length.

We also found that at any chain length of the triblock copolymers, the central beads *B* of triblock copolymers preferentially segregated into homopolymers *B*_n_ bulk phase, whereas the end beads *A* of triblock copolymers segregated to the homopolymers *A*_n_ bulk phase [Figure 3c and Figure S4], which indicates that the triblock copolymers form a “hairpin” type of conformation at the interface. In comparison with the diblock copolymers, these hairpin-type triblock copolymers cause the reduction in the mixing entropy and the interfacial tension, which results in the enhanced stability of the interfaces.

**Figure 5:**
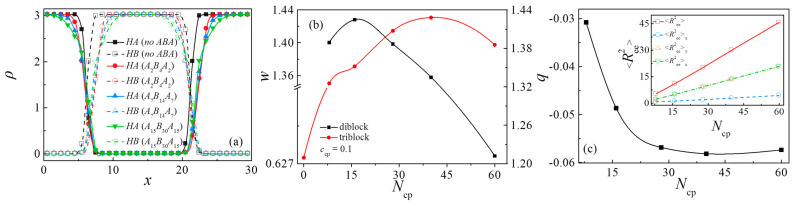
(**a**) Density profiles of homopolymer beads and (**b**) interface thickness in ternary blends at copolymer concentration of *c*_cp_ = 0.1. (**c**) Orientation parameter of triblock copolymers as a function of their chain length *N*_cp_. The inset indicates the mean-squared, end-to-end distance <*R*_ee_^2^> and the three principal components <*R*_ee_^2^>_x_, <*R*_ee_^2^>_y_, <*R*_ee_^2^>_z_.

Figure 5a,b show the variations in density profiles of homopolymers *A*_n_ and *B*_n_ and the interfacial width at different chain lengths of the diblock and triblock copolymers when *c*_cp_ = 0.1. The calculation of the interfacial width between the homopolymers *A*_n_ and *B*_n_ is according to the previous work of Guo et al. [4]. We extract the width *w* by fitting the function tanh ((*x + d*)/*w*) to the profile (ρ^A^(*x*) − ρ^B^(*x*))/ρ(*x*) across each interface, where *d* stands for the interface center shift along the *x*-directions. For comparison, the density profiles and the interfacial width of the pure *A*_n_/*B*_n_ binary blends without copolymers are also provided [the filled and vacant squares in Figure 5a, and the first point (i.e., *N*_cp_ = 0) in Figure 5b].

Our simulation indicates that the density profile gaps for the homopolymers and the interfacial widths of the ternary blends are broader than the pure duality homopolymer blends because of the occupation of triblock copolymers at the interfaces. As the chain length of the triblock copolymers increases from *N*_cp_ = 8 to 60, the density of beads *A* and *B* in homopolymers near the center of the interface exhibits an increase, whereas the density of beads *A* and *B* in homopolymers near the edge of the interface exhibits a decrease (as illustrated in Figure S3). With the triblock copolymers, the interfacial widths exhibit an increase of *N*_cp_ (the chain length of triblock copolymers) from 8 to 40, and decrease slightly as *N*_cp_ further increases from *N*_cp_ = 40 to 60 (the red filled dots in Figure 5b). However, with the diblock copolymers, as the *N*_cp_ increases from *N*_cp_ = 8 to 16, the interfacial widths increase slightly, and as *N*_cp_ further increases from *N*_cp_ = 16 to 60, the interfacial widths decrease rapidly (the black filled squares in Figure 5b). We inferred that the decrease in the interfacial widths for the cases with the longer chain length of copolymers is related to the distribution of copolymer beads. This phenomenon becomes more obvious in the diblock copolymer system. As the chain length of the copolymers increases to *N*_cp_ = 60, beads *A* and *B* of the copolymers tend to penetrate the corresponding homopolymer phase, which results in the reduction of copolymer segregation at the interface.

Figure 5c shows the orientation parameter *q* and the mean-square, end-to-end distance <*R*_ee_^2^> (as well as <*R*_ee_^2^>_x_, <*R*_ee_^2^>_y_, <*R*_ee_^2^>_z_) of the triblock copolymers at different triblock copolymer chain lengths. We found that as *N*_cp_ increases, <*R*_ee_^2^> and its components <*R*_ee_^2^>_y_, <*R*_ee_^2^>_z_ exhibit an increase, and <*R*_ee_^2^>_x_ is smaller than the components in *y* and *z* directions. The chain orientation parameter is *q* < 0 and decreases rapidly as *N*_cp_ increases from 8 to 28 with *c*_cp_ = 0.1, but as *N*_cp_ increases from 28 to 60, the chain orientation parameter remains almost unchanged.

### 3.4. Effect of Concentration of Triblock Copolymers

**Figure 6:**
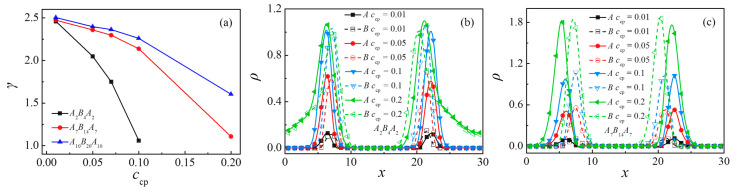
Interfacial tension as a function of triblock copolymer concentration at chain length *N*_cp_ = 8, 28, 40 (**a**). Density profiles of beads *A* and *B* of the triblock copolymer along the *x*-axis as a function of triblock copolymer concentration at copolymer chain length (**b**) *N*_cp_ = 8, and (**c**) *N*_cp_ = 28.

We further investigate the effects of the concentration of triblock copolymers *c*_cp_ on the interfacial properties. Figure 6a shows the interfacial tension of the ternary blends as a function of the triblock copolymer concentration *c*_cp_. The interfacial tension decreases as the concentration of the triblock *c*_cp_ increases. These results are in qualitative agreement with the experimental findings of Wagner et al. [9]. We also find that the interfacial tension has a larger value for longer triblock copolymer *N*_cp_ = 40 at the fixed copolymer concentration, which indicates that the shorter triblock copolymers show better performance in reducing the interfacial tension of the blends.

**Figure 7:**
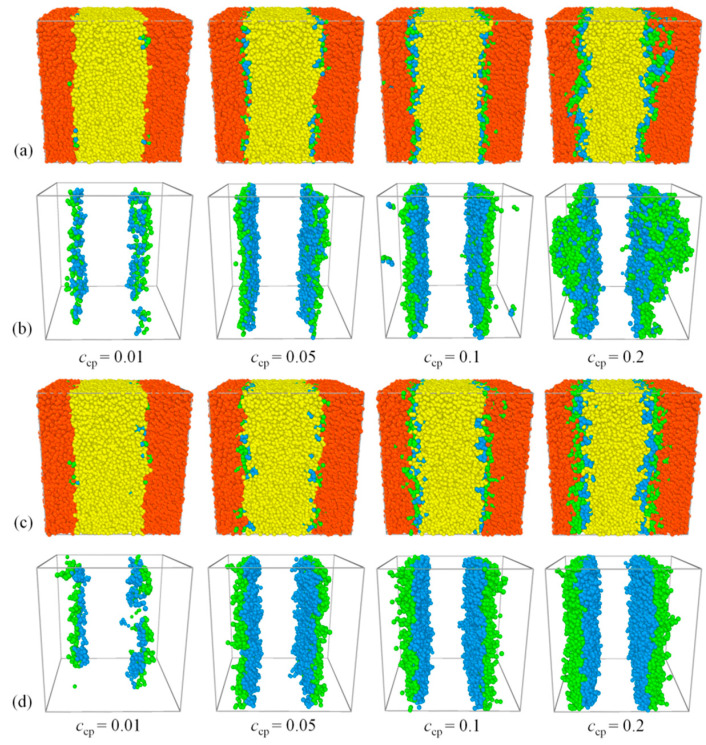
Morphology snapshots for ternary mixtures at different triblock copolymer concentrations. Compositions are (**a**) *A*_8_/*A*_2_*B*_4_*A*_2_/*B*_8_; (**b**) *A*_2_*B*_4_*A*_2_; (**c**) *A*_8_/*A*_7_*B*_14_*A*_7_/*B*_8_; (**d**) *A*_7_*B*_14_*A*_7_. Red and yellow spheres represent bead *A* and bead *B* of homopolymers, and green and blue spheres represent beads *A* and *B* of copolymers.

Figure 6b,c show the simulated density profiles (ρ) of beads *A* and *B* of triblock copolymers, with *N*_cp_ = 8 and *N*_cp_ = 28 as a function of *c*_cp_, respectively. In accord with Figure 6b,c, Figure 7 shows the morphology snapshots of ternary blends. In the case of *N*_cp_ = 8, as *c*_cp_ increases from 0.1 to 0.2, the densities of the *A*, *B* (Figure 6b), and beads *A* + *B* (Figure S5a) of triblock copolymer near the center of the interface only increases slightly, the triblock copolymer segregation in the homopolymers bulk *A*_n_ is enhanced, and the interface is no longer flat (Figure 7b). However, for the case of *N*_cp_ = 28, as the concentration of the triblock copolymer increases from *c*_cp_ = 0.01 to 0.2, the densities of the *A*, *B* [as illustrated in Figure 6c], and beads *A* + *B* [as illustrated in Figure S5b] of triblock copolymers at the interface exhibit significant increases, all the triblock copolymers enrich at the interface; i.e., no triblock copolymers segregate at the bulk of homopolymers and the interface always remains flat [as illustrated in Figure 7c,d]. Further, our simulation indicates that at a higher copolymer concentration of *c*_cp_ = 0.5, the ternary blends form BME structures rather than the macroscopic phase separation structure (as illustrated in Figures S6 and S7).

**Figure 8:**
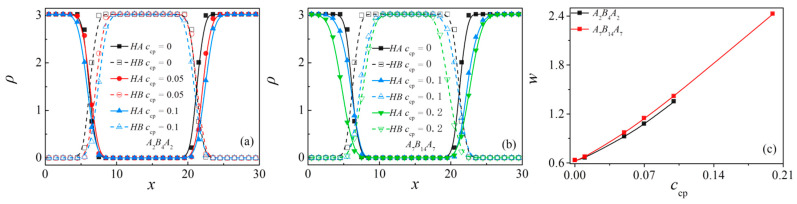
Density profiles of homopolymer beads along the *x*-axis as a function of triblock copolymer concentration at the copolymer chain length of (**a**) *N*_cp_ = 8 and (**b**) *N*_cp_ = 28, respectively. Interface thickness in ternary blends as a function of triblock copolymer concentration at the chain length of *N*_cp_ = 8, 28 (**c**).

The density profiles of beads *A* and *B* of the homopolymers and the interfacial width w along the *x*-axis as a function of triblock copolymer concentration at the copolymer chain length of *N*_cp_ = 8 (*c*_cp_ = 0 to 0.1) and *N*_cp_ = 28 (*c*_cp_ = 0 to 0.2) are shown in Figure 8a,b, respectively. It is shown that as the concentration of triblock copolymer increases, the density of homopolymer near the center of the interface decreases and the interfacial width *w* increases monotonically, which illustrates the decayed correlations between beads *A*_n_ and *B*_n_ of homopolymer with increasing the triblock copolymer concentration.

**Figure 9:**
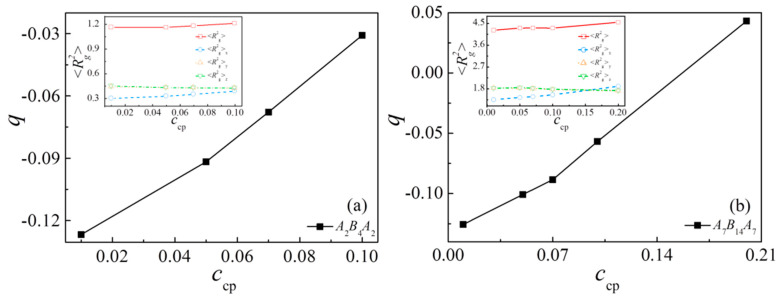
Orientation parameter of the triblock copolymers as a function of their chain length *N*_cp_. The inset indicates the mean-squared radii of gyration <*R*_g_^2^> and the three principal components (<*R*_g_^2^>_x_, <*R*_g_^2^>_y_ and <*R*_g_^2^>_z_) of <*R*_g_^2^>. (**a**) *N*_cp_ = 8 and (**b**) *N*_cp_ = 28, respectively.

Finally, we calculate the orientation parameter *q* and the mean-square radius of gyration <*R*_g_^2^> (as well as <*R*_g_^2^>_x_, <*R*_g_^2^>_y_, <*R*_g_^2^>_z_) of the triblock copolymers at different triblock copolymer concentrations in Figure 9a,b. We found that as the concentration increases, <*R*_g_^2^>_x_ also exhibits an increase. For the case of *N*_cp_ = 28 and *c*_cp_ = 0.2, the *x* component of <*R*_g_^2^> is greater than the components in *y* and *z* directions. In addition, the chain orientation parameter *q* increases monotonically as the concentration of the triblock copolymer increases. Combining the value of *q* from Figure 9a,b, we can see that: (1) the orientation parameter *q* exhibits a larger value for shorter triblock copolymer at the same concentration; (2) as the concentration of the triblock copolymer increases from *c*_cp_ = 0.1 to 0.2 with *N*_cp_ = 28, the chain orientation parameter *q* is greater than 0, which indicates that the triblock copolymer with higher concentration is more stretched along the *x*-direction, which is perpendicular to the interface.

## 4. Conclusions

We employed DPD simulation to study the interfacial properties of symmetric ternary *A*_n_/*A*_m_*B*_m_/*B*_n_ and *A*_n_/*A*_m/2_*B*_m_*A*_m/2_/*B*_n_ polymeric blends. We systematically explored the effects of composition, chain length, and copolymer concentration on the interfacial tension, the density of different beads near the interface, the interfacial width, and the detailed chain conformations in the polymeric blend.

Through the comparison between the interfacial tensions of the *A*_n_/*A*_m_*B*_m_/*B*_n_ and *A*_n_/*A*_m/2_*B*_m_*A*_m/2_/*B*_n_ blends, we found that the triblock copolymer is more effective in reducing the interfacial tension than the diblock copolymer, and the density of beads *A* + *B* of the triblock copolymers at the center of the interface is higher than that of the diblock. For the *A*_n_/*A*_m/2_*B*_m_*A*_m/2_/*B*_n_ polymeric blend systems, the addition of shorter triblock copolymer chains results in a more reduced interfacial tension and a higher density of copolymers at the center of the interface, which indicates the better performance of triblock copolymers as the compatibilizers compared to that of their counterparts with longer chain lengths. By elevating the concentration of the triblock copolymer, the density of the triblock copolymer increases, the interfacial width *w* enlarges, and the interfacial tension decreases; accordingly, the triblock copolymers are more stretched along the direction perpendicular to the interface.

Our simulations indicate that the efficiency of the added copolymer compatibilizers is strongly correlated with their microscopic architectures and chain properties. In this context, it would be necessary to systematically explore the effects of other molecular details on the interfacial and phase properties of blends composed of the triblock as the compatibilizers. Besides, given that ion-containing polymer mixtures serve as a new class of material that combines ionic conductivity with mechanical robustness [51], we also predict that study of the effects of copolymers (as compatibilizers) on ion-containing polymer/polymer or polymer/inorganic compound blends would contribute to the optimization of the polymer electrolytes used for new batteries and energy storage devices. Our work provides insights into the fundamental understanding of phase properties for polymer blends and the design of the copolymers as the ideal compatibilizer.

## Data Availability

The data presented in this study are available on request from the corresponding author.

## Supplementary Materials

The following are available online at https://www.mdpi.com/article/10.3390/polym13091516/s1. Figure S1: the radial distribution functions between (a) beads *A* and *B* [*g*_AB_(r)] from homopolymers *A*_n_ and *B*_n_, (b) beads *A* and *A* [*g*_AA_(r)] of all homopolymers *A*_n_, (c) beads *A* and *A* [*g*_AAintra_(r)] within homopolymers *A*_n_, and (d) beads *A* and *A* [*g*_AAinter_(r)] within different homopolymers *A*_n_ for the case *A*_8_/*A*_4_*B*_4_/*B*_8_ at different simulation times; Figure S2: the radial distribution functions between (a) beads *A* and *B* [*g*_AB_(r)] from homopolymers *A*_n_ and *B*_n_, (b) beads *A* and *A* [*g*_AA_(r)] of all homopolymers *A*_n_, (c) beads *A* and *A* [*g*_AAintra_(r)] within homopolymers *A*_n_, and (d) beads *A* and *A* [*g*_AAinter_(r)] with different homopolymers *A*_n_ for the case *A*_8_/*A*_2_*B*_4_*A*_2_/*B*_8_ at different simulation times; Figure S3: density profiles of beads *A* + *B* of the triblock copolymer along the *x*-axis as a function of triblock copolymer concentration at the copolymer chain length, (a) *N*_cp_ = 8, and (b) *N*_cp_ = 28; Figure S4: morphology snapshots of ternary blends in the simulation. (a) *N*_cp_ = 8 and *c*_cp_ = 0.5, (b) *N*_cp_ = 28 and *c*_cp_ = 0.5; Figure S5. Density profiles of beads *A* + *B* of the triblock copolymer along the *x*-axis as a function of triblock copolymer concentration at the copolymer chain length (a) *N*_cp_ = 8, and (b) *N*_cp_ = 28; Figure S6. Density profiles of beads *A* and *B* of the homopolymers and triblock copolymer along the *x*-axis at the copolymer chain length (a) *N*_cp_ = 8, and (b) *N*_cp_ = 28 with triblock copolymer concentration *c*_cp_ = 0.5; Figure S7. Representative morphology snapshots for ternary mixtures at triblock copolymer concentration *c*_cp_ = 0.5. The compositions are *A*_8_/*A*_2_*B*_4_*A*_2_/*B*_8_ for (a), and *A*_8_/*A*_7_*B*_14_*A*_7_/*B*_8_ for (b). The red and yellow spheres represent bead *A* and bead *B* of homopolymers, and the green and blue spheres represent beads *A* and *B* of the copolymers.

## References

[1] Wang J.F., Muller M., Wang Z.G. (2009). Nucleation in A/B/AB blends: Interplay between microphase assembly and macrophase separation. J. Chem. Phys..

[2] Chang K., Macosko C.W., Morse D.C. (2007). Ultralow interfacial tensions of polymer/polymer interfaces with diblock copolymer surfactants. Macromolecules.

[3] Retsos H., Anastasiadis S., Pispas S., Mays J., Hadjichristidis N. (2004). Interfacial Tension in Binary Polymer Blends in the Presence of Block Copolymers. 2. Effects of Additive Architecture and Composition. Macromolecules.

[4] Guo H.X., Cruz M.O. (2005). A computer simulation study of the segregation of amphiphiles in binary immiscible matrices: Short asymmetric copolymers in short homopolymers. J. Chem. Phys..

[5] Qian H.J., Lu Z.Y., Chen L.J., Li Z.S., Sun C.C. (2005). Dissipative particle dynamics study on the interfaces in incompatible A/B homopolymer blends and with their block copolymers. J. Chem. Phys..

[6] Kancharla S., Zoyhofski N.A., Bufalini L., Chatelais B.F., Alexandridis P. (2020). Association between Nonionic Amphiphilic Polymer and Ionic Suifactant in Aqueous Solutions: Effect of Polymer Hydrophobicity and Micellization. Polymers.

[7] Lemos T., Abreu C., Pinto J.C. (2020). DPD Simulations of Homopolymer-Copolymer-Homopolymer Mixtures: Effects of Copolymer Structure and Concentration. Macromol. Theory Simul..

[8] Russell T.P., Mayes A.M., Deline V.R., Chung T.C. (1992). Hairpin configurations of triblock copolymers at homopolymer interfaces. Macromolecules.

[9] Wagner M., Wolf B.A. (1993). Effect of block copolymers on the interfacial-tension between 2. Immiscible/homopolymers. Polymer.

[10] Jorzik U., Wagner M., Wolf B.A. (1996). Effect of block copolymer architecture on the interfacial tension between immiscible polymers. Prog. Colloid. Polym. Sci..

[11] Welge I., Wolf B.A. (2001). Reduction of the interfacial tension between ‘immiscible’ polymers: To which phase one should add a compatibilizer. Polymer.

[12] Harrats C., Fayt R., Jérme R., Blacher S. (2003). Stabilization of a cocontinuous phase morphology by a tapered diblock or triblock copolymer in polystyrene-rich low-density polyethylene/polystyrene blends. J. Polym. Sci. Pt. B Polym. Phys..

[13] Fayt R., Jerome R., Teyssie P. (1987). Characterization and control of interfaces in emulsified incompatible polymer blends. Polym. Eng. Sci..

[14] Dai K.H., Washiyama J., Kramer E.J. (1994). Segregation study of a bab triblock copolymer at the a/b homopolymer interface. Macromolecules.

[15] Hlavata D., Horak Z., Fort V. (1996). Localization of styrene-butadiene block copolymers in polystyrene/polypropylene blends. Polym. Eng. Sci..

[16] Xu Y., Thurber C.M., Macosko C.W., Lodge T.P., Hillmyer M.A. (2014). Poly(methyl methacrylate)-*block*-polyethylene-*block*-poly(methyl methacrylate) Triblock Copolymers as Compatibilizers for Polyethylene/Poly(methyl methacrylate) Blends. Ind. Eng. Chem. Res..

[17] Sun Z.Q., Zhang B., Bian X.C. (2015). Synergistic effect of PLA-PBAT-PLA tri-block copolymers with two molecular weights as compatibilizers on the mechanical and rheological properties of PLA/PBAT blends. RSC Adv..

[18] Zhao X.Y., Huang Y.J., Kong M.Q., Li G.X. (2018). Assessment of compatibilization efficiency of SEBS in the PP/PS blend. J. Appl. Polym. Sci..

[19] Peter C., Kremer K. (2010). Multiscale simulation of soft matter systems. Faraday Discuss..

[20] Shi A.C., Li B.H. (2013). Self-assembly of diblock copolymers under confinement. Soft Matter.

[21] Balazs A.C., Siemasko C.P., Lantman C.W. (1991). Monte-Carlo simulations for the behavior of multiblock copolymers at a penetrable interface. J. Chem. Phys..

[22] Wang Y.M., Li Y., Mattice W.L. (1993). Simulation of the adsorption of unsymmetric diblock copolymers at the interface between the 2 monomeric homopolymers. J. Chem. Phys..

[23] Schmid F., Müller M. (1995). Quantitative comparison of self-consistent-field theories for polymers near interfaces with monte-carlo simulations. Macromolecules.

[24] Müller M., Schick M. (1996). Bulk and interfacial thermodynamics of a symmetric, ternary homopolymer-copolymer mixture: A Monte Carlo study. J. Chem. Phys..

[25] Werner A., Schmid F., Binder K., Müller M. (1996). Diblock copolymers at a homopolymer-homopolymer interface: A Monte Carlo simulation. Macromolecules.

[26] Liu D.M., Duan X.Z., Shi T.F., Jiang F., Zhang H.Z. (2015). Monte Carlo Simulation of Effects of Homopolymer Chain Length on Interfacial Properties of A/AB/B Ternary Polymer Blends. Chem. J. Chin. Univ. Chin..

[27] Liu D.M., Dai L.J., Duan X.Z., Shi T.F., Zhang H.Z. (2015). Monte Carlo Simulation of Interfacial Properties in Homopolymer/Diblock Copolymer/Homopolymer Ternary Polymer Blends. Chem. J. Chin. Univ. Chin..

[28] Chen L., Xiao S., Zhu H., Wang L., Liang H. (2016). Shape-dependent internalization kinetics of nanoparticles by membranes. Soft Matter.

[29] Jiang Y., Zhang D., He L., Zhang L. (2016). Entropic Interactions in Semiflexible Polymer Nanocomposite Melts. J. Phys. Chem. B.

[30] Wang J.J., Li Z.Z., Gu X.P., Feng L.F., Zhang C.L., Hu G.H. (2012). A dissipative particle dynamics study on the compatibilizing process of immiscible polymer blends with graft copolymers. Polymer.

[31] Anastasiadis S.H., Gancarz I., Koberstein J.T. (1988). Interfacial-tension of immiscible polymer blends - temperature and molecular-weight dependence. Macromolecules.

[32] Zhu P.F., Li Y., Li Q.W. (2011). Mesoscopic Simulation of the Interfacial Behavior of Biosurfactant Rhamnolipids and the Synergistic Systems. Acta. Chim. Sin..

[33] Catarino Centeno R., Bustamante-Rendon R.A., Hernandez-Fragoso J.S., Arroyo-Ordonez I., Perez E., Alas S.J., Gama Goicochea A. (2017). Surfactant chain length and concentration influence on the interfacial tension of two immiscible model liquids: A coarse-grained approach. J. Mol. Model..

[34] Zhang Y.Z., Xu J.B., He X.F. (2018). Effect of surfactants on the deformation of single droplet in shear flow studied by dissipative particle dynamics. Mol. Phys..

[35] Liang X.P., Wu J.Q., Yang X.G. (2018). Investigation of oil-in-water emulsion stability with relevant interfacial characteristics simulated by dissipative particle dynamics. Colloid Surf. A-Physicochem. Eng. Asp..

[36] Wang S.Y., Yang S.W., Wang R.C. (2018). Dissipative particle dynamics study on the temperature dependent interfacial tension in surfactant-oil-water mixtures. J. Pet. Sci. Eng..

[37] Goodarzi F., Zendehboudi S. (2019). Effects of Salt and Surfactant on Interfacial Characteristics of Water/Oil Systems: Molecular Dynamic Simulations and Dissipative Particle Dynamics. Ind. Eng. Chem. Res..

[38] Goodarzi F., Kondori J., Rezaei N., Zendehboudi S. (2019). Meso- and molecular-scale modeling to provide new insights into interfacial and structural properties of hydrocarbon/water/surfactant systems. J. Mol. Liq..

[39] Huo J.H., Jiang H., Chen Z., Zhou J. (2018). Homoporous polymer membrane via forced surface segregation: A computer simulation study. Chem. Eng. Sci..

[40] Jiang H., Chen T.L., Chen Z., Huo J.H., Zhang L.Z., Zhou J. (2020). Computer Simulations on Double Hydrophobic PS-b-PMMA Porous Membrane by Non-solvent Induced Phase Separation. Fluid Phase Equilib..

[41] Groot R.D., Warren P.B. (1997). Dissipative particle dynamics: Bridging the gap between atomistic and mesoscopic simulation. J. Chem. Phys..

[42] Lin Y., Boker A., He J.B., Sill K., Xiang H.Q., Abetz C., Li X.F., Wang J., Emrick T., Long S. (2005). Self-directed self-assembly of nanoparticle/copolymer mixtures. Nature.

[43] Hong Z.H., Xiao N., Li L., Xie X.N. (2020). Investigation of nanoemulsion interfacial properties: A mesoscopic simulation. J. Food Eng..

[44] Zhang J.W., Chen L., Wang A.L., Yan Z.C. (2020). Dissipative Particle Dynamics Simulation of Ionic Liquid-Based Microemulsion: Quantitative Properties and Emulsification Mechanism. Ind. Eng. Chem. Res.

[45] Hoogerbrugge P.J., Koelman J. (1992). Simulating microscopic hydrodynamic phenomena with dissipative particle dynamics. Europhys. Lett..

[46] Koelman J., Hoogerbrugge P.J. (1993). Dynamic simulations of hard-sphere suspensions under steady shear. Europhys. Lett..

[47] Jananl S., Lisa M.H. (2018). Impact of ion content and electric field on mechanical properties of coarse-grained ionomers. J. Chem. Phys..

[48] Irving J.H., Kirkwood J.G. (1950). The statistical mechanical theory of transport processes .4. the equations of hydrodynamics. J. Chem. Phys..

[49] Hillmyer M.A., Maurer W.W., Lodge T.P., Bates F.S., Almdal K. (1999). Model bicontinuous microemulsions in ternary homopolymer block copolymer blends. J. Phys. Chem. B.

[50] Stoykovich M.P., Edwards E.W., Solak H.H., Nealey P.F. (2006). Phase behavior of symmetric ternary block copolymer-homopolymer blends in thin films and on chemically patterned surfaces. Phys. Rev. Lett..

[51] Liu B.L., Hu B., Du J., Cheng D.M., Zang H.Y., Ge X., Tan H.Q., Wang Y.H., Duan X.Z., Jin Z. (2021). Precise Molecular-Level Modification of Nafion with Bismuth Oxide Clusters for High-performance Proton-Exchange Membranes. Angew. Chem.Int. Edit..

