# Supplementary material for: Dissipative Particle Dynamics Study on Interfacial Properties of Symmetric Ternary Polymeric Blends
Source: Polymers (Basel). 2021 May 8;13(9):1516. doi: 10.3390/polym13091516 (PMC8125886; doi:10.3390/polym13091516)
Supplement: Supplementary file 1 [file polymers-13-01516-s001.zip › polymers-1174253-supplementary.pdf]

polymers

Article

Dissipative Particle Dynamics Study on Interfacial Properties of Symmetric Ternary Polymeric Blends

Dongmei Liu [1,*], Kai Gong [1], Ye Lin [1], Tao Liu [1,*], Yu Liu [1] and Xiaozheng Duan [2,3,*]

1 School of Science, North China University of Science and Technology, Tangshan, 063210, P. R. China; dmliu@ncst.edu.cn (D.L.); gongkai0524@163.com (K. G.); linye315317@163.com (Y.L.); liutaocreate@gmail.com (T.L.); vampireliu@outlook.com (Y.L.)

2 State Key Laboratory of Polymer Physics and Chemistry, Changchun Institute of Applied Chemistry, Chinese Academy of Science, Changchun, 130022, P. R. China; xzduan@ciac.ac.cn (X.D.)

3 State Key Laboratory of Molecular Engineering of Polymers, Department of Macromolecular Science, Fudan University, Shanghai, 200438, P. R. China; xzduan@ciac.ac.cn (X.D.)

* Correspondence: dmliu@ncst.edu.cn; Tel.: +86-315-8805860 (D.L.); liutaocreate@gmail.com ; Tel.: +86-315-8805860 (T.L.);xzduan@ciac.ac.cn; Tel.: +86-431-85262479 (X.D)

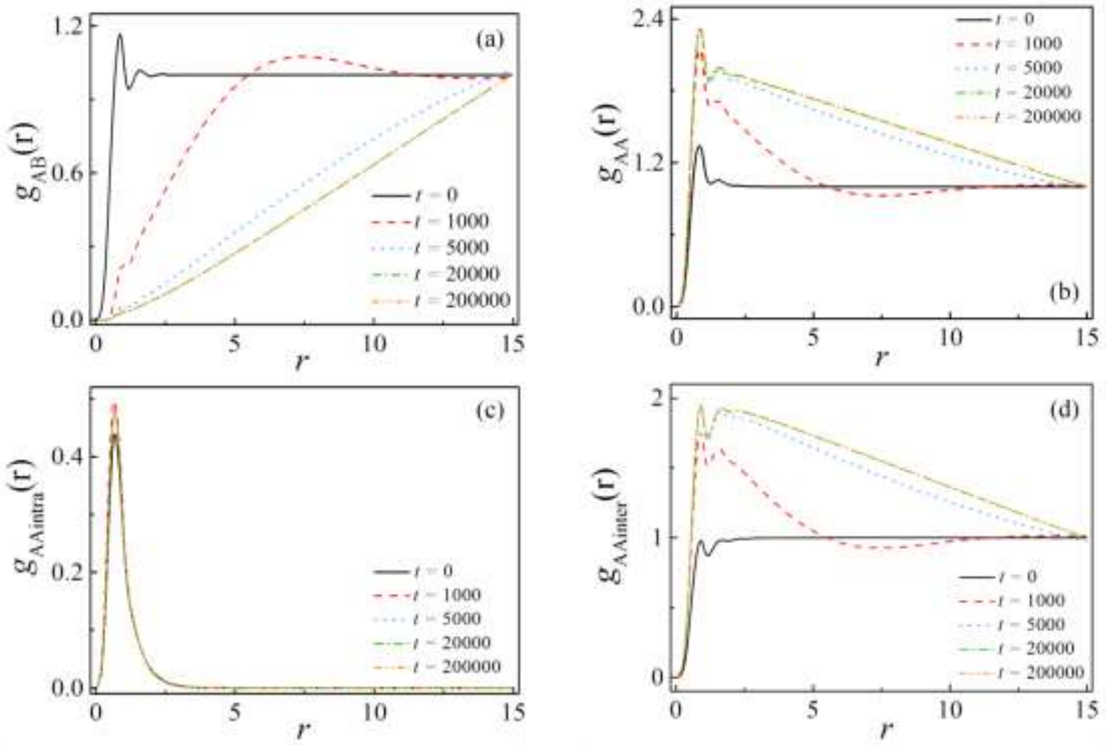

Figure S1. The radial distribution functions between (a) beads A and B [g_{AB}(r)] from homopolymers A_n and B_n, (b) beads A and A [g_{AA}(r)] of all homopolymers A_n, (c) beads A and A [$g_{AAintra}$(r)] within homopolymers A_n, and (d) beads A and A [$g_{AAinter}$(r)] within different homopolymers A_n for the case $A_8/A_4B_4/B_8$ at different simulation times. The copolymer concentration is set as $c_{cp} = 0.05$.

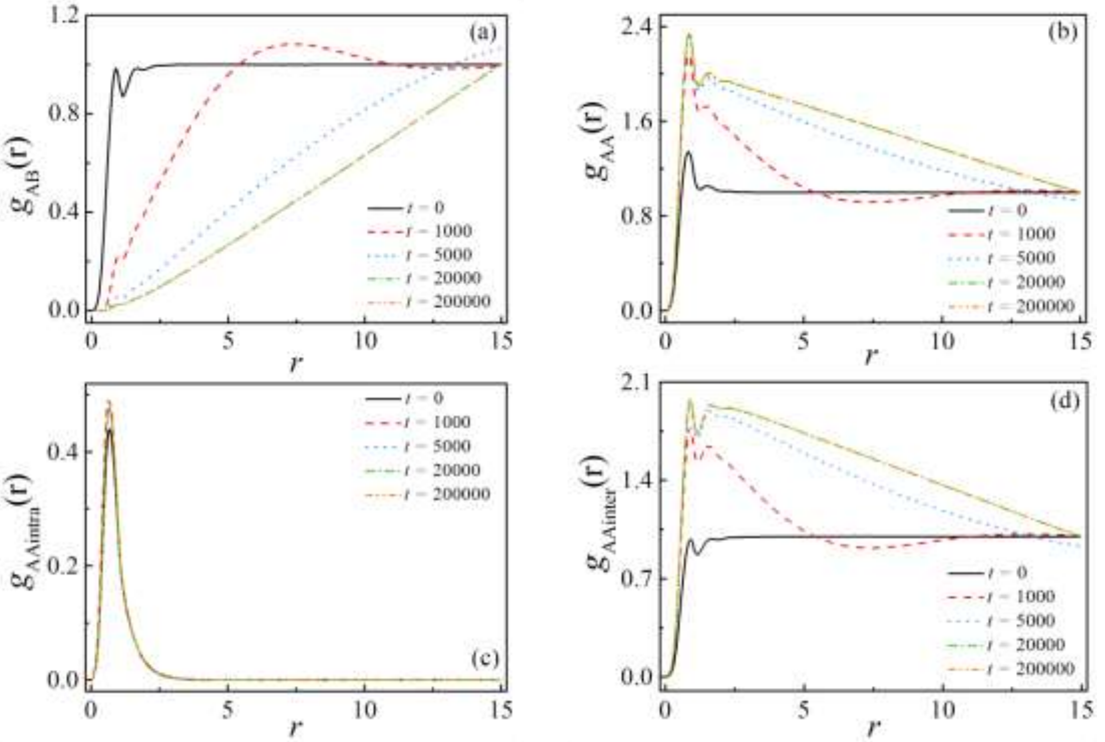

Figure S2. The radial distribution functions between (a) beads *A* and *B* [g_{AB}(r)] from homopolymers A_n and B_n, (b) beads *A* and *A* [g_{AA}(r)] of all homopolymers A_n, (c) beads *A* and *A* [$g_{AAintra}$(r)] within homopolymers A_n, and (d) beads *A* and *A* [$g_{AAinter}$(r)] with different homopolymers A_n for the case $A_8/A_2B_4A_2/B_8$ at different simulation times. The copolymer concentration is set as $c_{cp} = 0.05$.

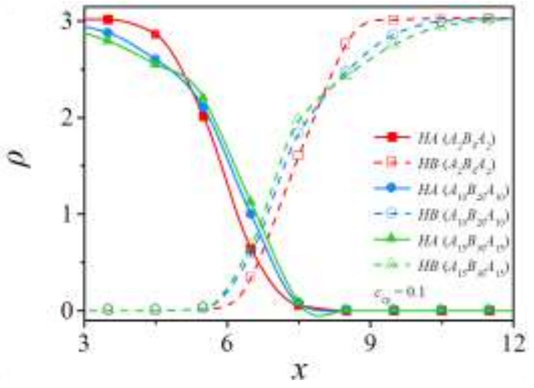

Figure S3. The zoom of Figure 5(a) at the interface.

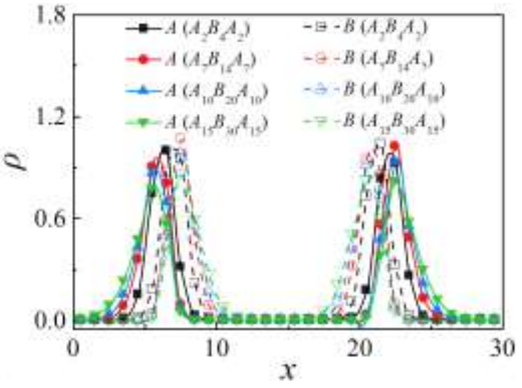

Figure S4. Density profiles of beads *A*, *B* of the triblock copolymer along the *x*-axis as a function of chain length of the copolymer at the copolymer concentration of $c_{cp} = 0.1$.

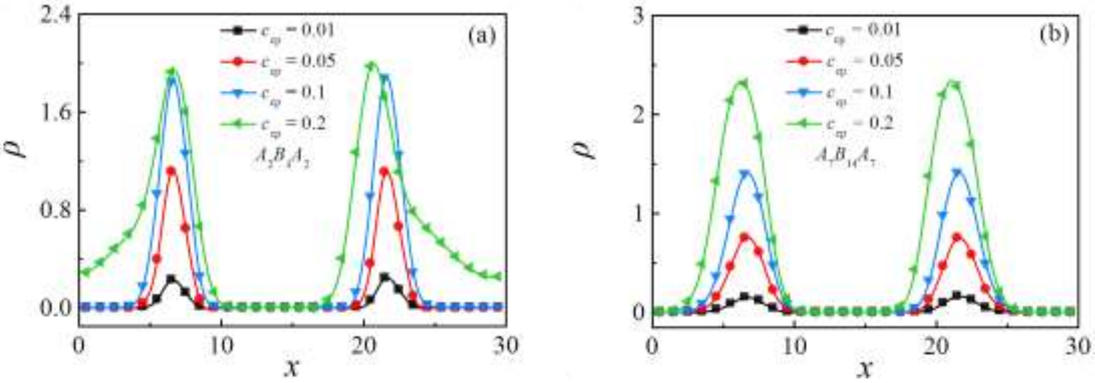

Figure S5. Density profiles of beads $A + B$ of the triblock copolymer along the x-axis as a function of triblock copolymer concentration at the copolymer chain length (a) $N_{cp} = 8$, and (b) $N_{cp} = 28$.

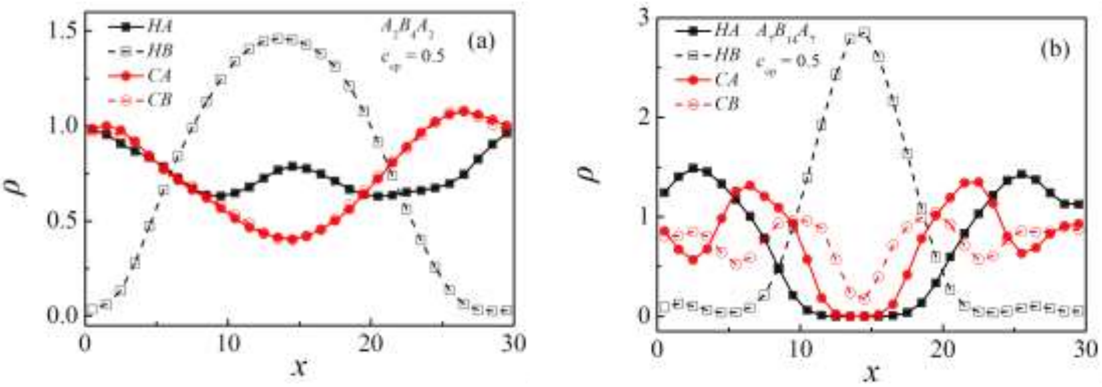

Figure S6. Density profiles of beads A and B of the homopolymers and triblock copolymer along the x-axis at the copolymer chain length (a) $N_{cp} = 8$, and (b) $N_{cp} = 28$ with triblock copolymer concentration $c_{cp} = 0.5$.

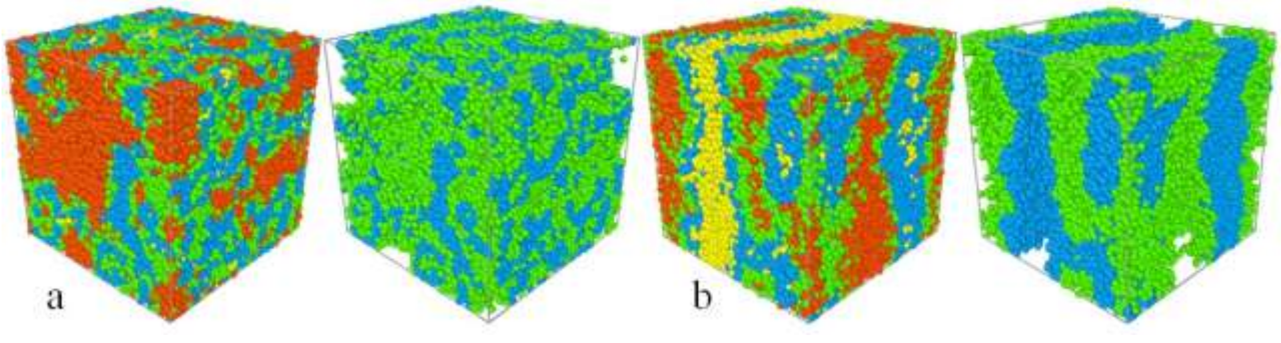

Figure S7. Representative morphology snapshots for ternary mixtures at triblock copolymer concentration $c_{cp} = 0.5$. The compositions are $A_8/A_2B_4A_2/B_8$ for (a), and $A_8/A_7B_{14}A_7/B_8$ for (b). The red and yellow spheres represent bead A and bead B of homopolymers, and the green and blue spheres represent beads A and B of the copolymers.
